# Putting together and taking apart: assembly and disassembly of the Rad51 nucleoprotein filament in DNA repair and genome stability

**DOI:** 10.15698/cst2018.05.134

**Published:** 2018-03-28

**Authors:** Tadas Andriuskevicius, Oleksii Kotenko, Svetlana Makovets

**Affiliations:** 1 Institute of Cell Biology, School of Biological Sciences, University of Edinburgh.

**Keywords:** homologous recombination, Rad51 filament, double-stranded DNA break, DNA repair, Rad51 regulation

## Abstract

Homologous recombination is a key mechanism providing both genome stability and genetic diversity in all living organisms. Recombinases play a central role in this pathway: multiple protein subunits of Rad51 or its orthologues bind single-stranded DNA to form a nucleoprotein filament which is essential for initiating recombination events. Multiple factors are involved in the regulation of this step, both positively and negatively. In this review, we discuss Rad51 nucleoprotein assembly and disassembly, how it is regulated and what functional significance it has in genome maintenance.

## INTRODUCTION

Homologous recombination (HR) involves exchange of genetic information, often between two different DNA molecules. This exchange requires physical interaction between the molecules and may lead to heritable genetic changes, contributing to biodiversity and higher evolutionary adaptability of species. Novel combinations of alleles arising in meiosis is a great example of genetic variability as a result of HR. On the other hand, physical linkage between chromosomes during meiotic HR is important for pairing and accurate segregation of homologous chromosomes in meiosis, thereby preventing aneuploidy and promoting genome integrity. In addition, HR machinery contributes to genome stability by playing a central role in stabilisation and restart of stalled replication forks as well as repair of DNA breaks routinely arising from broken replication forks, oxidative damage, etc. Because DNA breaks are part of normal cell physiology, unsurprisingly, defects in HR in humans lead to developmental disorders and cancer predisposition 1.

**Figure 1:**
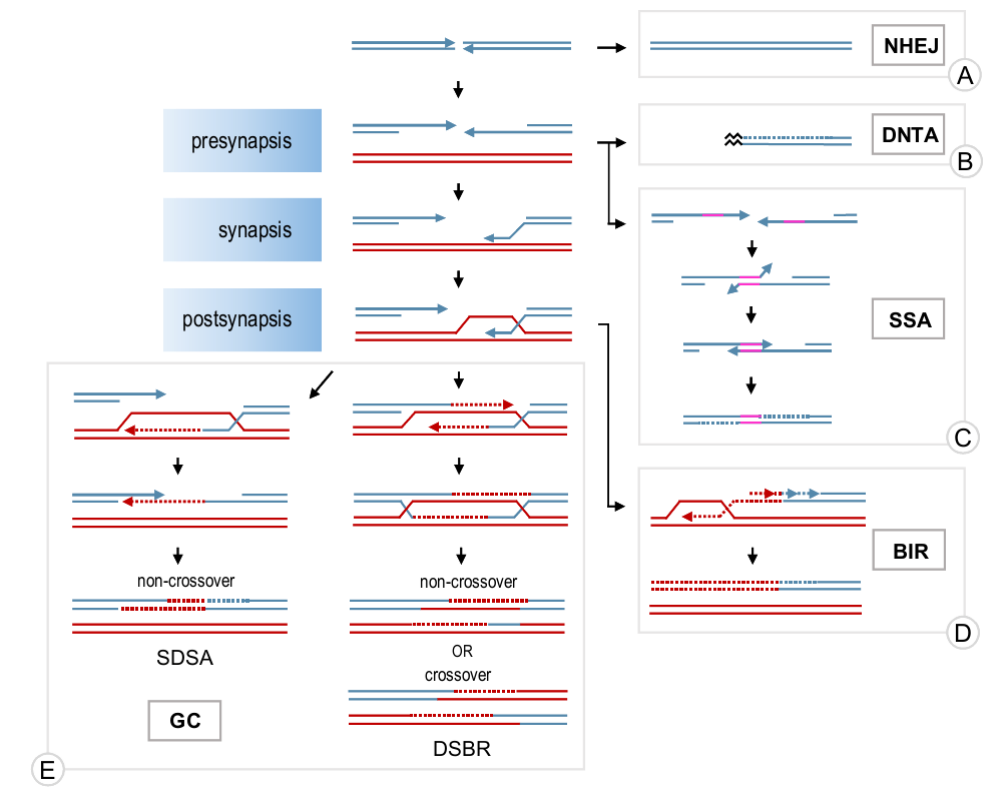
FIGURE 1: A general overview of DSB repair mechanisms. A DNA molecule with a DSB (blue lines) can either be repaired by **NHEJ (A)** or resected, thereby committing to HR. Sometimes, resected breaks can be healed by telomerase (**DNTA, B**) rather than repaired by HR but these events are rare. Resected DNA can be repaired by **SSA (C)** when there are direct repeats flanking the break (pink lines). Alternatively, resected ssDNA might invade a homologous donor molecule (red lines) enabling repair by **BIR (D)** or **GC (E)**. During **BIR,** the invading strand is extended and the newly synthesised DNA is displaced from the donor to act as a template for the second strand. **GC** can proceed via **SDSA** or **DSBR** pathways. During **SDSA,** the invading ssDNA is extended, displaced from the donor and annealed to the other end of the break. **DSBR** involves the extension of the invading strand, capture of the second end of the break and the resolution of the dHJ intermediate formed. Arrow heads indicate 3' ends of the DNA. Dotted lines show newly-synthesised DNA and their colours correspond to the DNA molecules that have been used as templates. The black zigzag represents the telomere. Abbreviations: NHEJ - non-homologous end joining; DNTA -* de novo *telomere addition; SSA - single-strand annealing; BIR - break-induced replication; GC - gene conversion; SDSA - synthesis-dependent strand annealing; DSBR - double-strand break repair.

A double-stranded DNA break (DSB) is often considered the most dangerous type of DNA damage as it disrupts the continuity of chromosomes and, if unrepaired, may lead to loss of genetic information and eventual cell death. Two major mechanisms are normally used to repair a DSB: non-homologous end joining (NHEJ) and HR (**Figure 1**). NHEJ involves ligation of the broken ends with little or no DNA processing around the break. It is efficient but often mutagenic due to small deletions or insertions at the damage locus 2,3,4. Occasionally, it can also lead to gross chromo-somal rearrangements due to illegitimate joining of DNA ends from different breaks or ligations of chromosome breaks to telomeres 5,6,7. HR includes a group of pathways sharing two features: i) they require intact homologous DNA sequences for the repair (called donor DNA) and ii) they stem from the same original step of extensive DSB resection around the break, which generates single-stranded DNA (ssDNA) necessary for HR pathways to operate. Although DSB repair is well-conserved among eukaryotes, the preference for the repair mechanism is cell cycle-dependent and species-specific 3,8,9. Occasionally, a DSB can be mistakenly recognised as a telomere and telomerase, the enzyme responsible for telomere extension, heals the break by adding a *de novo* telomere (**Figure 1**) 10. Although *de novo* telomere addition (DNTA) may stabilise the broken chromosome, it results in terminal deletions.

Depending on the nature of a DSB break, homology-dependent repair may proceed through gene conversion (GC), break-induced replication (BIR) or single-strand annealing (SSA; **Figure 1**). GC is used to repair a DSB when both ends of the break have homology to a donor DNA molecule and are available for the repair, whereas BIR is employed when only one end is present, for example, to restore broken replication forks 11,12. SSA does not involve external homology and can only be used if a DNA break is flanked by homologous repeats in direct orientation. SSA is mutagenic as any sequences between the homologies and one of the two copies of the repeated sequences are lost 13. However, SSA may act as a safeguard mechanism when other options are not available.

All three homology-dependent repair pathways begin with the resection of the 5’ end around a DSB to create ssDNA overhangs 14. SSA may occur at this point through annealing of the complementary sequences on the resected ends 13. GC and BIR require invasion of the resected DNA end into a donor molecule and a subsequent strand exchange where one strand of the donor molecule is displaced by the invading strand to form a D-loop. The invading strand is then extended using the homologous donor as a template 11,12. In the case of GC, only the area around the DSB is copied. GC may then proceed through a so-called synthesis-dependent strand annealing (SDSA) mechanism where the invading strand is displaced from the donor molecule and annealed to the sequences on the other end of the break (**Figure 1**). Alternatively, the second end of the break may be captured by the D-loop forming a double Holliday junction intermediate which can then be resolved by endonucleases or helicases (double-strand break repair pathway - DSBR) 11. In contrast to SDSA, which generates exclusively non-crossovers, the resolution of a double Holiday junction may lead to either a crossover or a non-crossover. In BIR, the donor molecule is replicated from the invasion site all the way to the telomere by conservative replication (**Figure 1**) 15. Both, GC and BIR may result in error-free repair if a sister chromatid is used as a donor. However, if the repair involves a homologous chromosome or an ectopic homology site, loss of heterozygosity or chromosomal rearrangements may occur 11,12,16.

The homology search and strand exchange reaction required for DSB repair by GC and BIR are catalysed by a recombinase called Rad51. Rad51 binds to resected ssDNA forming a Rad51-ssDNA filament (presynapsis) which then catalyses the search for homologous sequences and establishes a physical contact between the broken and donor molecules (synapsis) by invading the duplex donor DNA (**Figure 1**). As this step is absolutely required and is often rate-limiting in DSB repair, Rad51 assembly and disassembly plays an important role in modulating HR. In addition, disassembly of Rad51 filament is also required at the late stages of repair to restore the double-stranded structure of DNA 17. Recently, a novel role for Rad51 in protection and restart of stalled replication forks has emerged 18. Therefore, understanding the dynamics of Rad51 filaments is important for elucidating the molecular mechanisms of DNA repair and genome stability.

## RAD51 RECOMBINASE 

Rad51 was first genetically identified in 1974 in a screen for *Saccharomyces cerevisiae* mutants sensitive to ionising radiation 19. Since then, the involvement of Rad51 in genome integrity and dynamics has been well-characterised, defining it as one of the key enzymes required for HR 20,21,22,23,24,25. Rad51 is conserved among eukaryotes: Rad51 from vertebrates shares on average 74% protein sequence identity with fungi and plants while the human and mouse homologs are 99% identical 26. Rad51 belongs to the ancient RecA/RAD51 protein family which apart from the bacterial and archaeal orthologues, RecA and RadA respectively, includes members that have diverged from the original function and adapted to more specialised roles. For example, Dmc1 is a meiosis-specific equivalent of Rad51, whereas yeast Rad55 and Rad57 are Rad51 paralogues involved in Rad51 regulation 27,28,29,30. Although most eukaryotic orthologues are called Rad51 some, like the *Schizosaccharomyces pombe* orthologue Rhp51, may have a different name. For simplicity, these orthologues as a whole will be referred to as Rad51/RAD51 further in the review.

High protein sequence conservation suggests that the function of Rad51 is also conserved. Indeed, Rad51 deficient yeast and mouse trophoblast-like cells exhibit closely similar defects, including increased sensitivity to ionising radiation and chromosome loss, both of which can be explained by compromised DSB repair 19,25,31. However, mammalian cells appear to be more sensitive to the loss of RAD51: a homozygous gene deletion results in embryonic lethality and failure to establish *Rad51* null cell lines 31. This is also true for the chicken DT40 cells which accumulate spontaneous DNA DSBs in the absence of RAD51 and eventually die 32. The stronger severity of the *RAD51* null phenotype in vertebrates compared to yeasts suggests a greater reliance of these cells on conserved RAD51 activities, possibly due to the emerging role of RAD51 in preventing DNA damage during conventional replication 18 or the higher frequency of stochastic DSBs per cell cycle stemming from the larger genome sizes 31.

**Figure 2:**
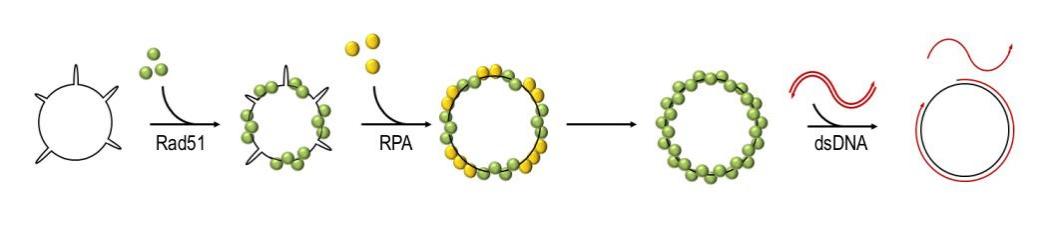
FIGURE 2: Rad51-catalysed strand exchange reaction. Circular ssDNA is pre-incubated with Rad51 to allow Rad51 binding to DNA without competition. RPA is then added to the reaction to remove secondary DNA structures. Nucleated Rad51 can replace RPA and form a functional continuous nucleofilament. When linear dsDNA is added Rad51 can catalyse the strand exchange between the double-stranded donor and the circular ssDNA.

Rad51 exists as a monomer in a solution but it can polymerise on both double-stranded DNA (dsDNA) and ssDNA in a cooperative manner and forms a right-handed nucleoprotein filament, in which the DNA is stretched and extended to facilitate homology search and base pairing 24,33,34,35,36,37. Unlike RecA, which has a very low affinity for dsDNA, Rad51 can bind both ssDNA and dsDNA* in vitro*, albeit with a preference for ssDNA 24,38,39,40. As required for its recombinase activity, Rad51 can bind two DNA molecules simultaneously, through its primary and secondary DNA binding sites. The primary binding site is responsible for the interactions with DNA during the formation of the filament and the double-stranded product in postsynapsis, while the secondary site is required for the capture of a donor molecule in presynapsis 41,42,43. Rad51 can carry out the strand-exchange reaction *in vitro* (**Figure 2**). This activity is strongly stimulated by addition of the ssDNA binding protein RPA (Replication Protein A) after Rad51 is pre-nucleated with ssDNA 40,44,45,46,47,48. The positive effect of RPA was postulated to result from the ability of RPA to remove secondary DNA structures which might impede the formation of continuous, functional Rad51 filaments 47,48,49.

Rad51 also binds ATP and hydrolases it in a DNA-dependent manner. The ATP binding is required for Rad51 activities 40,44,45,50,51. Rad51 mutants deficient in nucleotide binding are catalytically dead and cannot form extended nucleofilaments, while mutants that bind ATP but are incapable of hydrolysing it can perform the strand exchange reaction *in vitro* and partially complement *RAD51 *deletion *in vivo* when overexpressed 52,53. This demonstrates that only ATP binding is required for the essential Rad51 functions but ATP hydrolysis contributes to its full activity inside the cell. However, the role of Rad51 ATP hydrolysis is not completely understood. Possibly, it is important for the disassembly of the filament and recycling of Rad51 pool as *S. cerevisiae* Rad51, just like its bacterial orthologue RecA, shows decreased affinity for DNA when bound to ADP instead of ATP 54,55,56. Consistent with this hypothesis, human RAD51 bound to DNA shows lower subunit turnover when ATP hydrolysis is prevented 37,57. Alternatively, the ATP hydrolysis might promote the DNA annealing step during the strand exchange reaction: while the ATP-bound nucleofilament conformation is stiffer and more suitable for the initial step of separating donor DNA strands, the ADP-bound human RAD51 might promote annealing of the invading and donor strands 58. Interestingly, *in vitro* ATP hydrolysis by Rad51 is much slower than that by RecA. When bound to ssDNA, human and yeast orthologues hydrolyse 0.16 and 0.7 ATPs per minute per subunit respectively while RecA turns over 25-30 ATP molecules during that time 40,44,54,59. Therefore, the modulation of the ATPase activity of Rad51 *in vivo* by other proteins might have an important function in regulating Rad51 activity.

## POSITIVE REGULATORS OF RAD51 NUCLEOPROTEIN FILAMENT FORMATION

As mentioned above, DSB repair by GC and BIR requires a formation of Rad51 filament on resected DNA. However, resected DNA is much more readily coated by the ssDNA binding protein RPA, which is more abundant inside the cells and has a higher affinity for ssDNA than Rad51 60. As mentioned earlier, RPA stimulates Rad51-catalysed strand exchange *in vitro* when it is added after Rad51 has been assembled on ssDNA. However, if RPA is incubated with ssDNA before the Rad51 addition or both proteins are introduced into the reaction simultaneously, Rad51 filament formation and the subsequent strand exchange reaction are largely inhibited 46,48,61,62,63. This indicates that Rad51 and RPA compete for binding to ssDNA and that Rad51 cannot efficiently replace RPA bound to ssDNA. However, the RPA/Rad51 protein exchange can be facilitated by so called mediator proteins described below.

### Rad52 and BRCA2

**Figure 3:**
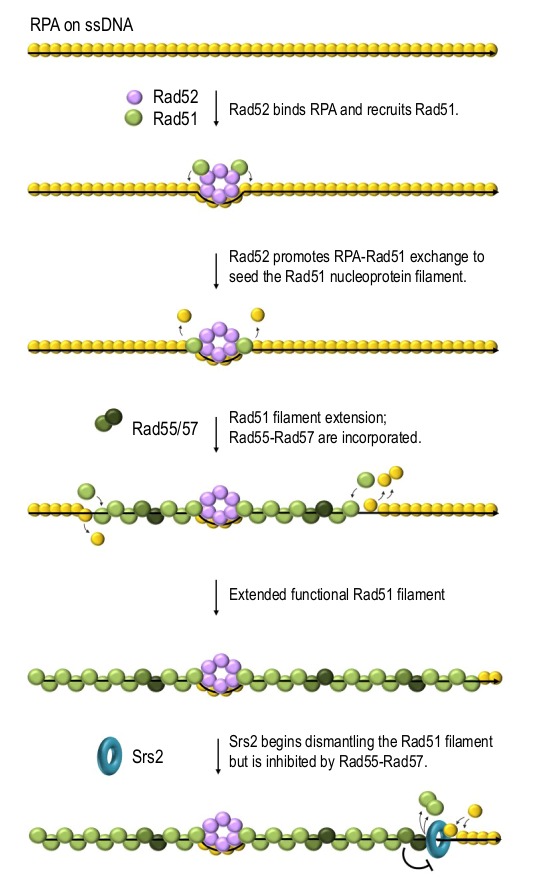
FIGURE 3: A model of Rad51 nucleofilament formation in budding yeast. ssDNA generated as a result of resection or strand separation is rapidly covered by RPA (yellow spheres). Rad52 (purple spheres) binds RPA-coated ssDNA, recruits Rad51 (green spheres) and facilitates RPA-Rad51 exchange in the vicinity, thereby promoting Rad51-ssDNA filament formation. Rad55-Rad57 dimers (dark-green spheres) are also incorporated into the Rad51 nucleofilament and stabilise it by providing additional protein-protein interactions as well as antagonising Rad51 removal by Srs2 (teal ring). Only the main regulators of *S. cerevisiae *Rad51 are shown.

Rad52 is a mediator which promotes Rad51 filament formation on RPA-coated ssDNA 46,48,62,63. Rad52 can physically interact with DNA, RPA and Rad51 and it has been postulated to promote the formation of Rad51 filaments by both recruiting Rad51 and helping it to replace RPA on ssDNA 46,64,65,66. Rad52 binds and stabilises RPA on ssDNA while promoting RPA replacement by Rad51 on the Rad52-surrounding DNA, thereby nucleating Rad51 filaments (**Figure 3**) 67. The N-terminus of Rad51 has been demonstrated to interact with the DNA binding domain of RPA and possibly promote RPA dissociation 68. This might allow the nucleated Rad51 to help free Rad51 monomers to compete for DNA binding, thereby extending the filament. This explanation is consistent with the recent* in vitro* reconstruction studies suggesting that RPA mainly inhibits the nucleation but not the elongation of Rad51 filaments 37,67. Rad52 also stabilises Rad51 filament via protein-protein interactions 69. *S. cerevisiae* cells lacking Rad52 are deficient in DSB repair by HR and cannot form detectable DNA damage-induced Rad51 foci suggesting that Rad51 filament assembly requires Rad52 *in vivo* 70,71,72.

Rad52 orthologues are also present in vertebrates. However, RAD52 deficient mice, chicken and human cells show only a slight defect in HR and remain resistant to DSB inducing factors 73,74,75. This is due to RAD52 playing a secondary role in recombination in mammals while another protein, BRCA2, mediates RAD51 filament formation 76,77. Like *S. cerevisiae* Rad52, BRCA2 enables RAD51 to perform the strand exchange reaction with RPA-coated ssDNA substrate *in vitro* 77. BRCA2 physically interacts with RAD51, RPA and DNA 78,79. BRCA2 has been proposed to stabilise RAD51 filaments *in vitro* by blocking ATP hydro-lysis by RAD51 77,80. In addition, one of the BRCA2-interacting proteins, BRCCIPβ, also interacts with RAD51 and induces a conformational change which facilitates the normally slow release of ADP from RAD51, thereby promoting nucleotide exchange and reversal of RAD51 to the active ATP-bound state 81,82. Another HR accessory factor, HOP2-MND1, can induce RAD51 conformational changes which enhance its ability to bind nucleotides and modulate the ssDNA-binding ability to promote the strand exchange reaction 83.

Interestingly, human RAD52 cannot substitute for BRCA2 in nucleating RAD51 on RPA-coated ssDNA *in vitro* but RAD52 depletion in BRCA2 deficient cells results in synthetic lethality accompanied by a further decrease in HR and a reduction in RAD51 foci induced by ionising radiation 77,84. This implies that although BRCA2 is the main RAD51 mediator in human cells, RAD52 activity in promoting the assembly of the RAD51 filament is significant enough to keep BRCA2-deficient cells viable.

### Rad51 paralogues

Yeast Rad51 paralogues Rad55 and Rad57 are two other key mediators. They form a stable heterodimer which physically interacts with Rad51 and can alleviate the RPA-dependent inhibition of Rad51-catalysed strand exchange reaction *in vitro* 30. The phenotype of the *rad55* *rad57* double mutant is identical to those of individual deletions 85. Mutants lacking Rad55 are sensitive to DNA-damaging agents, deficient in DSB repair by HR and show impaired localisation of Rad51 to an unrepairable DSB suggesting that Rad51 filament assembly is defective in the absence of Rad55-Rad57 70,86. The *rad51-I345T* mutation results in a Rad51 protein with increased affinity for DNA and the ability to overcome the inhibitory effect of RPA during the strand exchange reaction *in vitro*. *rad51-I345T* also partially suppresses the DNA damage sensitivity of *rad55*Δ and *rad57*Δ cells but it cannot compensate for the loss of the Rad52 mediator activity 85. Rad55-Rad57 works downstream of the Rad52-dependent Rad51 recruitment to ssDNA and possibly stabilises Rad51 filament through protein-protein interactions, which might maximise the probability that a nucleation event will result in a successful assembly of a functional Rad51 nucleofilament. Rad55-Rad57 has been also proposed to enhance the stability of Rad51 filaments by antagonising Srs2 helicase (described below) 29 which dislodges Rad51 from ssDNA (**Figure 3**) 87,88. Consistently, Rad51-I345T which partially compensates for the loss of Rad55-Rad57 is harder for Srs2 to strip from ssDNA *in vitro* 89 and the DNA damage sensitivity of cells lacking the Rad55-Rad57 complex can be suppressed by a deletion of *SRS2* 87,90,91.

Five Rad51 paralogues have been described in vertebrates: RAD51B, RAD51C, RAD51D, XRCC2 and XRCC3 27,28. They all function in the same pathway as BRCA2 and the depletion of any one of them results in a decreased efficiency of HR in human cells 92. Phylogenetic studies suggest that XRCC2 and RAD51D are yeast Rad55 and Rad57 orthologues respectively, although some studies place Rad57 most closely related to XRCC3 28,93,94,95. The Rad51 paralogs form two stable complexes: one is called BCDX2 and consists of RAD51B, RAD51C, RAD51D, and XRCC2 while the other one - CX3 - is made of RAD51C and XRCC3 96. The DNA damage induced formation of RAD51 foci is decreased in human cells depleted of RAD51D, RAD51C and XRCC2 but not XRCC3 suggesting that BCDX2 complex is required for efficient Rad51 filament formation, while CX3 is dispensable for this process 92,97. In agreement with this, the CX3 complex has been shown to act downstream of Rad51 filament assembly and participate in the resolution of Holliday junctions 98.

### Shu complex

Another factor that participates in Rad51 filament assembly is the Shu complex. In *S. cerevisiae,* it consists of the Rad51 paralogues Csm2 and Psy3 which form a heterotetramer along with Shu1 and Shu2 99,100. Unlike Rad52 and Rad55-Rad57, the Shu complex is not essential for DSB repair by HR *in vivo* 99*.* However, deletions of the individual genes encoding the Shu complex components do lead to defects in Rad51 foci formation 100. Csm2 physically interacts with Rad55 and bridges the Shu complex to Rad51 101. The Csm2-Psy3 dimer binds DNA and can enhance the Rad51-catalysed strand exchange reaction with RPA-coated substrates *in vitro*, in a Rad52- and Rad55-Rad57-dependent manner 101. Although Shu1 and Shu2 are not required for this activity* in vitro*, *shu1*Δ results in elevated Srs2-dependent Rad51 filament disassembly *in vivo *102. Furthermore, Shu2 physically interacts with Srs2 suggesting that the Shu complex might promote Rad51 filament assembly by both direct interactions with Rad51 and inhibiting the Srs2-dependent disassembly of the filament 103.

A Shu2 orthologue - SWS1 - has been found in humans and shown to interact with another protein, SWSAP1. This interaction mutually stabilises the two proteins and the SWS1-SWSAP1 complex can bind ssDNA. SWSAP1 was also found to physically interact with RAD51, RAD51B, RAD51C, RAD51D and XRCC3 while SWS1 can bind to RAD51D and XRCC3 104. Depletion of either SWS1 or SWSAP1 results in decreased formation of DNA damage-induced RAD51 foci suggesting that the function of the Shu complex is conserved from yeast to humans 104,105.

### Rad54

Other factors, although less significant, have been implicated in promoting Rad51 nucleofilament formation. Rad54/RAD54 is an ATP-dependent translocase which can bind Rad51/RAD51 and DNA simultaneously and plays an important role in synaptic and postsynaptic events. While a catalytically-dead translocase is deficient in DNA strand invasion, it is fully functional in stabilising Rad51 filaments 106,107,108. When Rad51 is assembled on dsDNA, Rad54 can strip it in an ATP-dependent manner 109,110. Therefore Rad54 promotes Rad51 filament formation on ssDNA by both stabilising Rad51 binding to ssDNA and inhibiting its association with dsDNA. However, cell cycle dependent phosphorylation of Rad54 can convert it into a negative regulator of Rad51, by enabling a Rad54-dependent removal of Rad51 at HR loci in G2 111.

### Swi5-Srf1 (Sae3-Mei5)

Sae3 and Mei5 are meiosis-specific mediators of Rad51 and Dmc1 filament formation in *S. cerevisiae* 112. However, their conserved orthologues in other organisms, including *S. pombe*, mice and humans (Swi5/SWI5 and Sfr1/SFR1 respectively), participate in the assembly of mitotic Rad51/RAD51 filament 113,114,115. Swi5/SWI5 and Sfr1/SFR1 form a stable complex with a 1:1 stoichiometry and can directly interact with Rad51/RAD51 114,115,116. *S. pombe* Swi5-Sfr1 stimulates the *in vitro* Rad51-dependent strand exchange reaction 117. Furthermore, the formation of Rad51/RAD51 foci after exposure to ionising radiation is decreased in both *sfr1*Δ *S. pombe* and SRF1 depleted human cells, pointing towards a defect in the assembly of Rad51 113,114. *S. pombe* Swi5 and Sfr1 proteins form an elongated structure *in vitro* which fits in the helical groove of the presynaptic filament, thereby suggesting a hypothetical mechanism for Swi5-Sfr1 action during the assembly of Rad51 116.

### INO80

It is important to note that Rad51 assembles on DNA in a context of chromatin. Not surprisingly, an evolutionally conserved nucleosome remodelling complex INO80 has been linked to the formation of the presynaptic filament 118,119,120,121. Disruption of the INO80 remodelling complex in budding yeast and human cells results in a decreased efficiency of HR. Rad51 accumulation on resected DNA is also reduced pointing to a defect in the formation of the nucleofilament. This phenotype can be largely suppressed by a removal of the H2A.Z histone variant suggesting that H2A.Z has an inhibitory effect on the assembly of Rad51 and that INO80 complex is required to remove it from damaged chromatin 118,119,122,123. However, the exact mechanism of how H2A.Z inhibits Rad51 filament formation is unknown. Although DNA resection is also affected in cells lacking INO80 118,119, the H2A.Z removal suppresses the defect of Rad51 filament assembly but it does not compensate for the resection defect 119. As HR efficiency is also largely restored in cells lacking both INO80 and H2A.Z, the main function of INO80 in HR is likely to be the facilitation of Rad51 nucleofilament formation 118,119.

## NEGATIVE REGULATION OF RAD51-DNA BINDING

### Rad51 removal from undamaged dsDNA

As mentioned above, Rad51 can bind both ssDNA and dsDNA *in vitro* 24,39. While Rad51 binding to ssDNA is essential for the strand exchange reaction *in vitro*, pre-coating dsDNA with Rad51 inhibits the formation of the product 45,109. *In vivo*, Rad51 cellular pools are limited and Rad51 binding to dsDNA depletes the pool of free monomers leaving fewer of them available for the repair 70. Thus, Rad51 binding to undamaged chromatin can impede Rad51-dependent recombination and may result in genome instability and chromosome loss 110,124.

In *S. cerevisiae*, non-damage-associated DNA binding of Rad51 is cytologically undetectable as it is actively antagonised by three SWI2/SNF2 family DNA translocases - Rad54, Rdh54 and Uls1 124. Both Rad54 and Rdh54 remove Rad51 from dsDNA in an ATP-dependent manner *in vitro* 109,125. This activity requires the N-terminal parts of Rad54 and Rdh54 which have been shown to interact with Rad51 but the exact mechanism of Rad51 displacement is unknown 126,127. Rad54 has been further demonstrated to enable the strand exchange reaction *in vitro*, even when both ssDNA and the donor dsDNA are covered with Rad51 109.

Rdh54 might be the main player in the removal of Rad51 from undamaged chromatin as the lack of this protein but not the other two translocases results in accumulation of spontaneous non-damage-associated Rad51 foci 124. However, deletion of all the three translocase-coding genes results in a more severe phenotype suggesting that Rad54 and Uls1 can partially substitute for Rdh54 124,128. Consistent with the *in vitro* evidence, inactivation of the ATPase activity in any of the three proteins results in the same phenotype as in the corresponding deletion mutants, further highlighting the need for the Rdh54 and Rad54 translocase activities in the removal of Rad51 from dsDNA; Uls1 is likely to operate in a similar way 124.

Rad54 homologs are well-conserved among eukaryotes, with human cells containing two known proteins - RAD54 and RAD54B 129. Like its yeast counterpart, human RAD54 can remove RAD51 from dsDNA *in vitro *110. Simultaneous depletion of both RAD54 and RAD54B results in accumulation of RAD51 on chromatin in human tumour cells 110. The fact that both proteins need to be depleted to reveal the phenotype suggests that they are redundant and that RAD54B also may have the ability to remove RAD51 from dsDNA. This suggests that the function of the discussed SWI2/SNF2 translocases in the regulation of the RAD51 cellular pool might be conserved from yeasts to humans.

### Suppression of HR at replication forks via inhibition of Rad51 filament formation

DNA synthesis in eukaryotes strongly depends on PCNA (Proliferating Cell Nuclear Antigen), a homotrimeric protein which forms a ring around the DNA and acts as a tool belt holding different components of the replication machinery: polymerases, ligases, nucleases, helicases, etc. 130. Some of these enzymes, such as DNA polymerases, are associated with PCNA at the fork almost all the time while others can be recruited as needs for their activity at specific loci arise. For example, Pif1 family helicases are recruited to hard-to-replicated loci 131. Replication fork barriers (tightly bound proteins, G-rich DNA regions, highly expressed genes, etc) cause replication fork pausing which often leads to accumulation of ssDNA, followed by recruitment of Rad52 and Rad51, thereby creating an opportunity for the DNA at the fork to be involved in unwanted HR. In budding yeast, these potentially mutagenic events are prevented by the Srs2 helicase which has been shown to disassemble the Rad51 nucleoprotein filament by dislodging Rad51 from ssDNA 87,88. Srs2 is recruited to replication forks through a direct binding of the SUMO-interacting motif (SIM) and the PCNA-interacting peptide box in the C-terminus of Srs2 to a SUMOylated PCNA 132,133,134. Once recruited to replication forks, Srs2 removes Rad51 and prevents potentially deleterious unscheduled HR events that might occur when replication fork progression is slowed down or paused by replication barriers. Disruption of this regulatory mechanism by mutations in *SRS2* leads to a hyper-recombination phenotype (increased mitotic recombination) 135,136. However, when replication forks are stalled due to a damaged template, Rad51 recruitment to the replication fork might be desirable in order to bypass the DNA lesion via the template switching mechanism 137. In this case, local Srs2 levels might be decreased through targeting the fork-bound Srs2 for proteasomal degradation 138. In addition, PCNA can be unloaded from stalled replication forks by the Elg1-containing RFC complex, thereby eliminating the PCNA-dependent binding of Srs2 to the fork 138.

Recent advances in understanding the role of RAD51 at replication forks in higher eukaryotes suggest an additional layer of regulatory mechanisms modulating RAD51 activities during replication. In human cells, a depletion of either BRCA2 or RAD51 results in under-replication and cell cycle arrest in the subsequent G1 phase 139. The current understanding of replication considers stalled replication forks as part of normal cell physiology and fork reversal as a mechanism stabilising the forks 18. RAD51 has been proposed to participate in both fork reversal 140 and protection of these forks from excessive DNA degradation by nucleases 141,142. A reversed replication fork is a four-way dsDNA junction, with one of the four branches terminating in a one-ended DSB which can serve as an entry point for the break resection machinery. BRCA2-dependent recruitment of RAD51 to a partially resected reversed fork has been proposed to inhibit further DNA degradation 142,143. On the other hand, RAD51 accumulation at replication forks is counteracted by a newly-identified ssDNA-binding protein RADX which prevents fork collapse due to excessive activity of RAD51 144. Interestingly, deleting *RADX* restores fork protection in BRCA2-deficient cells 144 suggesting that a fine balance between the positive and negative regulation of RAD51 at the forks by BRCA2 and RADX respectively is required for the genome stability maintenance during replication. Rad51 was further demonstrated to physically interact with the primase Polα, possibly by recruiting it to stalled replication forks and promoting their restart 142. Rad51 also prevents the degradation of nascent DNA at ssDNA gaps which might form behind replication forks due to a damaged DNA template. Normally, these gaps are small and might be undetectable but accumulation of extensive ssDNA tracks behind replication forks has been observed in the absence of Rad51 binding to chromatin in *Xenopus* egg extracts. The accumulation of these ssDNA gaps may be suppressed by the inhibition of the Mre11 nuclease activity, further supporting the role of Rad51 in the protection of nascent DNA against the degradation by nucleases 141.

The role of yeast Rad51 in fork protection is less understood but some close similarities to the findings in higher eukaryotes have been found: *RAD51* deletion in *S. cerevisiae* leads to accumulation of ssDNA gaps at forks and behind them. Rad51 and Rad52 localise to the forks during replication and are required for post-replicative DNA repair via HR 145. Therefore, the replication-associated functions of Rad51 might be conserved in eukaryotes.

### Disassembly of Rad51 nucleoprotein filament during DSB repair

On one hand, disassembly of Rad51 presynaptic filaments during DSB repair may play a role in limiting excessive recombination events. On the other hand, the same activity plays pro-recombination role as it is required at the late stages of repair to ‘clean up’ postsynaptic DNA in order to enable recruitment of PCNA and the rest of the replication machinery to re-synthesize resected DNA 17. The Srs2 helicase has now been implicated in both functions and therefore Srs2-dependent disassembly of Rad51 filament previously considered inhibitory to HR, also has a pro-recombination role as discussed below.

PCNA is recruited to DNA by Replication Factor C (RFC) which recognises primer-template junctions and loads PCNA on dsDNA 146,147,148. The RFC-PCNA complex can bind primer-template DNA junctions and in general has a significant affinity to ssDNA *in vitro*. The latter can be inhibited by the addition of RPA, which enhances the specificity of PCNA loading to the junctions 148,149,150. RPA physically interacts with RFC and stimulates PCNA loading onto DNA *in vitro* 17,151,152,153. In contrast, Rad51 inhibits PCNA loading but this inhibition can be suppressed by either increasing the concentrations of RPA, which competes with Rad51 for ssDNA binding, or by addition of the Srs2 helicase which disassembles Rad51 filaments, thereby promoting RPA binding to DNA and the consequential PCNA recruitment via RPA-RFC interactions 17,88,152.

Srs2 can remove RPA, Rad52 and Rad51 from ssDNA *in vitro* 55,87,88,89,154. Srs2 can physically interact with Rad51 and although *S. cerevisiae* Srs2 is capable of removing human RAD51 *in vitro*, the efficient clearance of ssDNA from Rad51 depends on species-specific interactions between Srs2 and Rad51, as well as ATP hydrolysis by Rad51 and Srs2 translocase activity 55,89. It has been suggested that Srs2 allosterically activates ATP hydrolysis in Rad51 monomers, thereby decreasing their affinity for DNA. Srs2 translocation is postulated to be important for the processivity and positioning of the helicase, which might be required to make appropriate contacts with the successive Rad51 monomers 55. In addition, a tandem assembly of Srs2 monomers appears to be important for the efficient disassembly of Rad51 filaments 89. On the other hand, the rate of Rad51 stripping is also influenced by the strength of the Rad51-ssDNA interaction: the amino acid substitutions which increase the stability of Rad51 on ssDNA due to either increased affinity for ssDNA or inability to hydrolyse ATP both slow down the rates of Rad51 removal by Srs2 89.

Importantly, Srs2 has been proposed to promote SDSA by disrupting D-loops in a manner that requires its ATPase activity, Rad51-interacting domain, SIM and PCNA-interacting peptide box 136,155,156,157. *SRS2* deletion leads to an elevated frequency of crossovers during the repair of an induced DSB 155.

It is not known in detail how Srs2 is brought to DNA repair sites but its localisation is independent of the SIM which is required for Srs2 recruitment to stalled replication forks via interaction with SUMOylated PCNA 133. The Srs2-ΔC (1-860) mutant protein lacking the Rad51-interacting region of Srs2 has a greatly impaired Rad51 clearance activity *in vitro*, mainly due to a decreased loading of mutant Srs2 on Rad51-ssDNA filaments 55,89. This suggests that the Rad51-interacting region of Srs2 promotes Srs2 association with the presynaptic filament 89. However, Srs2 has been shown to localise to repair sites even in the absence of Rad51 133. Furthermore, the *srs2-*Δ*C* (1-860) allele can suppress the DNA re-synthesis defects arising from the lack of Rad51 removal in *srs2*Δ cells 17. These observations suggest that there might be a Rad51-independent way to recruit Srs2 to repair sites *in vivo*. Consistently, it has been recently demonstrated that Srs2 can directly bind heteroduplex DNA joints *in vitro* 89.

**Figure 4:**
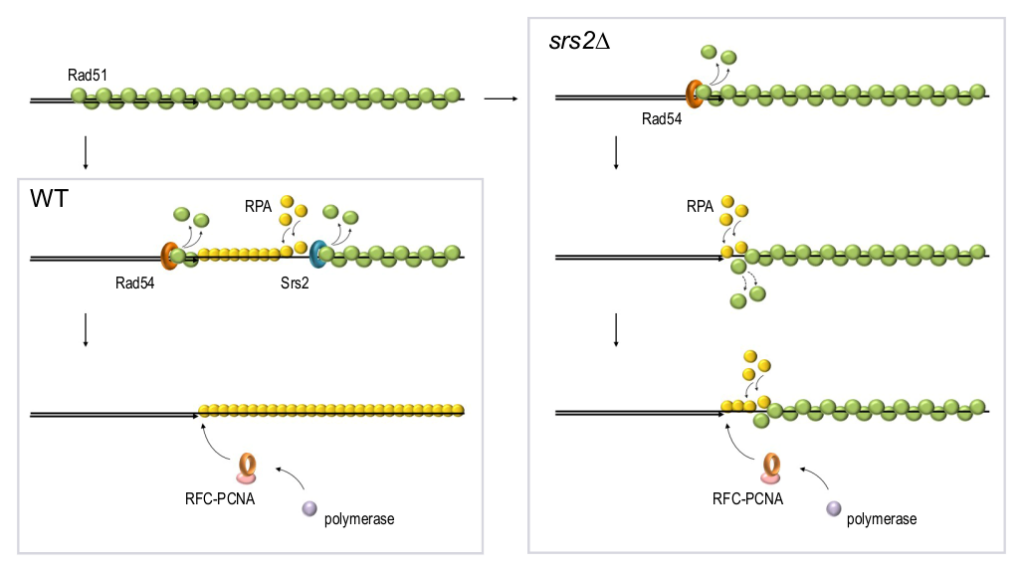
FIGURE 4: A hypothetical model for the complementing roles of Rad54 and Srs2 in Rad51 removal at dsDNA-ssDNA junctions. In wild-type cells, Srs2 (teal ring) and Rad54 (orange ring) remove Rad51 from ssDNA and dsDNA respectively to allow RFC-PCNA (pink ellipsoid and light-brown ring) to access the dsDNA-ssDNA junction and recruit DNA polymerase (light-purple sphere). In the absence of Srs2, Rad54 removes Rad51 from dsDNA and either directly or indirectly promotes Rad51 dissociation from ssDNA at the junction.

Inability to disassemble Rad51 filaments at the repair sites in *srs2*Δ cells leads to accumulation of ssDNA gaps, persistence of the DNA damage signalling and failure to complete DNA repair 17,158,159. These ssDNA gaps stem from a defect in re-synthesis of resected DNA which is likely required to terminate DNA resection. Although the re-synthesis is impaired in *srs2*Δ, the reconstitution of dsDNA during DSB repair can be observed in *srs2*Δ, albeit at a slower rate 17. This could be explained by a sufficiently high stochastic exchange between Rad51 and RPA on ssDNA. Alternatively, there might be other yet unidentified proteins involved in Rad51 removal during the late stages of repair. Our recent experiments indicate that Rad54 might be such a protein as the loss of both Srs2 and Rad54 almost completely blocks re-synthesis of resected DNA during DSB repair (Andriuskevicius and Makovets, unpublished results). Our current hypothesis is that while Srs2 removes Rad51 from ssDNA, Rad54 might be disrupting the extension of the Rad51 filament at the neighbouring dsDNA region (**Figure 4**). In the absence of Srs2, the Rad54-dependent removal of Rad51 from dsDNA might extend into the ssDNA. Alternatively, stripping of Rad51 from dsDNA by Rad54 might promote stochastic dissociation of Rad51 from the ssDNA at the junction and vice versa: dislodging of Rad51 by Srs2 from ssDNA may destabilise Rad51 bound to the dsDNA at the junction (**Figure 4**). This hypothesis is consistent with the observations that stochastic Rad51 dissociation from DNA is higher for the monomers at the end of the filament than for the internal ones 37,160. The dissociation of Rad51 from the dsDNA at the junction might be required not only for the recruitment of PCNA but also to make the 3’end accessible for DNA polymerases in order to initiate DNA polymerisation, both during re-synthesis of resected DNA and when extending the invaded DNA strand in a D-loop 161. This might explain why the post-invasion DNA synthesis during BIR in *srs2*Δ is also affected 17. To summarise, in yeast Rad54 and Srs2 might be complementing each other in disassembly of Rad51 filaments while being partially redundant in Rad51 removal from the ssDNA-dsDNA junction due to the intrinsic features of the Rad51 filament. Indirect evidence suggest that RAD54 might have a similar function in vertebrates 111,162.

A clear homolog of Srs2 has not been found in higher eukaryotes but other proteins have the ability to destabilise RAD51 nucleofilaments. G2-induced phosphorylation of Rad54 in *Xenopus* enables it to remove Rad51 from sites of HR 111. Human helicases FBH1, BLM, RECQL5 and FANCJ have been demonstrated to dislodge RAD51 from ssDNA in an ATP-dependent manner *in vitro* 163,164,165,166. Another protein called PARI also has the ability to remove RAD51 from ssDNA *in vitro*. Although PARI does not have an ATPase activity, the PARI-mediated disassembly of the nucleofilament is dependent on ATP hydrolysis by RAD51 suggesting that PARI, like Srs2, can stimulate RAD51 ATPase activity 167. This also supports the idea that ATP hydrolysis destabilises human RAD51 filament. Similarly to Srs2, PARI also functions at replication forks where it is recruited via PCNA and PCNA SUMOylation facilitates this recruitment 167. Mammalian cells lacking FBH1, BLM, RECQL5 or PARI exhibit hyper-recombination phenotypes suggesting the relevance of these proteins to the negative control of recombination *in vivo* 165,167,168,169,170.

Human cells might also be able to remove RAD51 from DNA at DNA damage sites via ubiquitination 162. The RFWD3 E3 ubiquitin ligase can physically interact with RAD51 and polyubiquitinate it both *in vitro* and *in vivo*. The RFWD3-dependent ubiquitination of RAD51 results in its degradation by proteasome. Cells depleted of RFWD3 or expressing an ubiquitination-deficient RAD51 variant show decreased turnover of RAD51 at repair sites, suggesting that the ubiquitination might destabilise RAD51 in the filament by targeting it for degradation. Although the exact stage of HR involving RFWD3-dependent regulation is unknown, it is proposed to function after the formation of RAD51 nucleofilament, and perhaps, even downstream of postsynapsis. If RFWD3 were destabilising RAD51 at the presynaptic stage and working as an anti-recombinase, the HR frequency would have been increased when RAD51 ubiquitination was prevented. However, the opposite has been observed in cells expressing ubiquitination-defective RAD51 mutant protein, suggesting that RFWD3-assisted RAD51 removal is important for the progression of HR at the later stages, possibly during the postsynaptic strand extension 162. Interestingly, decreased RAD54 chromatin loading was observed in RFWD3 deficient human cells after DNA damage, suggesting that RAD51 ubiquitination and RAD54 loading might be functionally linked 162.

It is important to emphasise that Rad51 filament is formed on resected DNA breaks independently of whether the repair will proceed by HR or by some other mechanism, for example, SSA or DNTA. Whichever route the repair takes, eventually the resected DNA is to be re-synthesised and converted back to dsDNA, in order to complete the repair and switch off the DNA damage signalling. Although Rad51 is not required for DSB repair via either SSA or DNTA, the Srs2-dependent activity on Rad51 is necessary for these mechanisms to be efficient 17. The results on DNTA are particularly interesting: they suggest that DSB healing by telomerase normally happens on resected DNA ends, although DNTA is dramatically increased in mutants with deficient resection 171,172. The evidence for DNTA and BIR raises a possibility that any DNA repair mechanisms that involve generation of ssDNA might be affected by unwanted Rad51 filament formation if the ssDNA is persistent long enough for the filament to assemble. Therefore, Rad51 removal by Srs2 or other means might be required not only during DSB repair but have a broader function in DNA repair.

## REGULATION OF RAD51 FILAMENT DYNAMICS THROUGH POST-TRANSLATIONAL MODIFICATIONS

Rad51 functions are regulated through post-translational modifications (PTMs) of not only Rad51 but also of the positive and negative regulators of Rad51 nucleoprotein filament assembly. The purpose of most of these modifications falls into one of the three categories: i) cell cycle dependent restrictions on the activity of a factor; ii) upregulation of DNA repair in cells with DNA damage; and iii) regulation of protein turnover at repair sites. Homology-based repair is limited to S-G2, mostly due to the cell cycle dependent phosphorylation required to activate the resection endonucleases upon entry in the S phase. This might explain why Rad52 localisation to DNA depends on the CDK1 activity 173,174 although CDK1-dependent phosphorylation of Rad52 has been detected in high-throughput experiments 175 and therefore Rad52 might be also directly regulated by CDK1. SUMOylation is involved in regulation of Rad52 too: it leads to stabilisation of the protein *in vivo* 176 while decreasing its affinity to ssDNA 177. In yeast, both Rad51 and Rad55 are phosphorylated in response to DNA damage and these PTMs are required for cell survival upon DNA damage induction 178,179,180. Activation of the DNA damage response in yeast as well as mammals leads to RPA SUMOylation 181,182,183 which increases its interaction with RAD51, possibly through the recently identified SIM within RAD51 184. Both RAD51 and BRCA2 are deubiquitinated in response to DNA damage 185,186: deubiquitination of BRCA2 leads to its stabilisation 186 while deubiquitinated RAD51 increases its binding to BRCA2 185. In turn, RAD51 ubiquitination might be important for RAD51 removal at the later stages of repair possibly by RAD54 162, as discussed above. The removal of Rad51 from repair foci in G2 in *Xenopus* is promoted by cell cycle specific phosphorylation of Rad54 111.

The role of multiple PTMs in regulation of Srs2 is well-documented. CDK1-dependent phosphorylation at the C-terminus promotes the interaction of Srs2 with Mre11 187, counteracts its SUMOylation and directs Srs2 to unwinding D-loops, thereby promoting DSB repair by SDSA 188. Although this phosphorylation controls the localisation of the Srs2 to D-loops, neither the elimination of the phosphorylation nor *SRS2* deletion has an effect on Rad51 presence at the site of strand invasion, suggesting that Srs2 acts downstream of the invasion step 188. The C-terminus of Srs2 also plays an important role in regulation of its function at replication forks. As mentioned above, the C-terminus of Srs2 contains a SIM which is required for Srs2 interaction with PCNA at replication forks. However, the C-terminus also contains SUMOylation sites and Srs2 SUMOylation inhibits its interaction with PCNA 189. Therefore, PTMs of Srs2 regulate both its anti-recombinase role at replication forks and its pro-recombination function in SDSA. The C-terminus is not required for the role of Srs2 in Rad51 removal during re-synthesis of resected DNA 17. However, the shortest functional C-terminal truncation Srs2 (1-860) retains two out of the seven identified CDK1 sites which might be important for this function. In summary, regulation of HR in general and Rad51 filament assembly in particular through PTM provides an additional layer of mechanisms to boost Rad51 activities in response to DNA damage, fine-tune them depending on the type of DNA damage and cell cycle stage as well as enables cells to regulate Rad51 and its interaction with DNA and other proteins as the repair progresses.

## CONCLUSION

The importance of recombinases in genome stability and diversity has been appreciated since the early days of molecular biology. Classical biochemistry and crystallography followed by more recently emerged single-molecule analysis and cryo-electron microscopy have provided molecular insights into their structure and function at the molecular level. We have also learned that numerous accessory proteins are required for a recombinase to operate *in vivo*; a whole set of enzymes built around it comprises HR machinery. The complexity of regulatory mechanisms governing this machinery has been becoming more and more obvious with the increasing number of discoveries of PTMs which modulate the components of HR machinery and their regulators. Rad51 filament formation is one of the most critical steps in HR and its regulation, both positive (filament assembly) and negative (disassembly) are required for efficient DNA repair. The newly-emerging role of recombinases at stalled replication forks adds another angle to understanding biological significance of their regulation. The sophistication of this regulatory network, based on the multitude of inputs and connections to other cellular pathways, increases from prokaryotes to yeast to higher eukaryotes, thereby underlying the importance of the fine-tuning of recombinase activities for cell survival and adaptive evolution of species.

## Abbreviations

BIR: break-induced replication
DNTA: de novo telomere addition
DSB: double-stranded DNA break
dsDNA: double-stranded DNA
GC: gene conversion
HR: homologous recombination
PTM: post-translational modification
RFC: Replication Factor C
RPA: Replication Protein A
SDSA: synthesis-dependent strand annealing
SIM: SUMO-interacting motif
SSA: single-stranded annealing
ssDNA: single-stranded DNA
